# Differential Proteomics of Cardiovascular Risk and Coronary Artery Disease in Humans

**DOI:** 10.3389/fcvm.2021.790289

**Published:** 2022-02-04

**Authors:** Ele Ferrannini, Maria Laura Manca, Giulia Ferrannini, Felicita Andreotti, Daniele Andreini, Roberto Latini, Marco Magnoni, Stephen A. Williams, Attilio Maseri, Aldo P. Maggioni

**Affiliations:** ^1^Consiglio Nazionale Delle Ricerche (CNR) Institute of Clinical Physiology, Pisa, Italy; ^2^Department of Clinical and Experimental Medicine, University of Pisa, Pisa, Italy; ^3^Department of Medicine Solna, Karolinska Institutet, Stockholm, Sweden; ^4^Institute of Cardiology, Fondazione Policlinico Universitario Gemelli, Istituto di Ricovero e Cura a Carattere Scientifico (IRCCS), Rome, Italy; ^5^Centro Cardiologico Monzino, Istituto di Ricovero e Cura a Carattere Scientifico (IRCCS), Milan, Italy; ^6^Department of Clinical Sciences and Community Health, Cardiovascular Section, University of Milan, Milan, Italy; ^7^Mario Negri Institute of Pharmacological Research-Istituto di Ricovero e Cura a Carattere Scientifico (IRCCS), Milan, Italy; ^8^Istituto di Ricovero e Cura a Carattere Scientifico (IRCCS) Ospedale San Raffaele and Università Vita-Salute San Raffaele, Milan, Italy; ^9^Clinical Research and Development, SomaLogic Inc., Boulder, CO, United States; ^10^Heart Care Foundation, Florence, Italy; ^11^Associazione Nazionale Medici Cardiologi Ospedalieri (ANMCO) Research Center, Heart Care Foundation, Florence, Italy

**Keywords:** coronary artery disease, cardiovascular risk factors, proteomics, atrial myosin regulatory light chain 2, protein shisa-3 homolog

## Abstract

**Background:**

Proteomics of atypical phenotypes may help unravel cardiovascular disease mechanisms.

**Aim:**

We aimed to prospectively screen the proteome of four types of individuals: with or without coronary artery disease (CAD), each with or without multiple risk factors. Associations with individual risk factors and circulating biomarkers were also tested to provide a functional context to the protein hits.

**Materials and Methods:**

The CAPIRE study (ClinicalTrials.gov Identifier: NCT02157662) is a cross-sectional study aimed at identifying possible new mechanisms promoting or protecting against atherothrombosis. Quantification (by aptamer technology), ranking (using partial least squares), and correlations (by multivariate regression) of ~5000 plasma proteins were performed in consecutive individuals aged 45–75 years, without previous cardiovascular disease, undergoing computed tomography angiography for suspected CAD, showing either >5/16 atherosclerotic segments (CAD^+^) or completely clean arteries (CAD^−^) and either ≤ 1 risk factor (RF^+^) or ≥3 risk factors (RF^−^) (based on history, blood pressure, glycemia, lipids, and smoking).

**Results:**

Of 544 individuals, 39% were atypical (93 CAD^+^/RF^−^; 120 CAD^−^/RF^+^) and 61% typical (102 CAD^+^/RF^+^; 229 CAD^−^/RF^−^). In the comparison with CAD^+^/RF^−^ adjusted for sex and age, CAD^−^/RF^+^ was associated with increased atrial myosin regulatory light chain 2 (MYO) and C-C motif chemokine-22 (C-C-22), and reduced protein shisa-3 homolog (PS-3) and platelet-activating factor acetylhydrolase (PAF-AH). Extending the analysis to the entire cohort, an additional 8 proteins were independently associated with CAD or RF; by logistic regression, the 12-protein panel alone discriminated the four groups with AUC_ROC_'s of 0.72–0.81 (overall *p* = 1.0e^−38^). Among them, insulin-like growth factor binding protein-3 is positively associated with RF, lower BMI, and HDL-cholesterol, renin with CAD higher glycated hemoglobin HbA_1c_, and smoking.

**Conclusions:**

In a CCTA-based cohort, four proteins, involved in opposing vascular processes (healing vs. adverse remodeling), are specifically associated with low CAD burden in high CV-risk individuals (high MYO and C-C-22) and high CAD burden in low-risk subjects (high PS-3 and PAF-AH), in interaction with BMI, smoking, diabetes, HDL-cholesterol, and HbA_1c_. These findings could contribute to a deeper understanding of the atherosclerotic process beyond traditional risk profile assessment and potentially constitute new treatment targets.

## Introduction

Coronary artery disease (CAD) is estimated to affect >200 million people worldwide (1). The currently accepted main risk factors for CAD remain those of the Framingham set, i.e. male sex, family history of premature CAD, age, smoking, diabetes, hypertension, hypercholesterolemia and HDL-cholesterol (2). Additional markers (e.g. troponin-T, C-reactive protein, and N-terminal pro-B-type natriuretic peptide) have proven useful in patients with acute myocardial infarction (MI), heart failure or myocardial hypertrophy, but have been shown to carry little incremental predictive value for chronic CAD (3). Ideally, new markers should also constitute potential treatment targets, but in recent years no novel molecule was identified in the context of CAD.

Cardiac ischemic events may develop when CAD reaches a critical threshold. However, there are patients with severe CAD who do not develop ischemic events; conversely, individuals with minimal CAD may do so. Similar exceptions to the accepted paradigm include patients with diffuse CAD with a low cardiovascular risk factor (RF) profile and others with multiple RFs who develop only mild or no CAD. While such “outliers” represent only 15–20% of patients experiencing an acute coronary event (4), they nevertheless offer a unique model to search for unknown mechanisms predisposing to CAD beyond traditional RFs; conversely, individuals witout significant CAD but many RFs may have unknown protective factors that impede or delay CAD.

The Coronary Atherosclerosis in outlier subjects: Protective and novel Individual Risk factors Evaluation (CAPIRE) study was designed to explore this opportunity by prospectively segregating RFs and CAD (assessed by coronary computed tomography angiography, CCTA) into two outlier groups of subjects, whose event rates are being followed in time (5). The rapidly evolving technology of proteomics provides a refined tool for developing new diagnostic and therapeutic algorithms (6). High-performance platforms can screen thousands of proteins that may serve as biomarkers, eventually to be included in multi-parametric models of risk assessment, and/or serve as indicators of disease pathways or of therapeutic effectiveness (7).

Here we report the findings obtained by applying large-scale proteomics to the entire cohort of subjects in the CAPIRE study, with a special focus on the two outlier groups, RF^−^/CAD^+^ and RF^+^/CAD.

## Methods

### Study Design

CAPIRE (ClinicalTrials.gov Identifier: NCT02157662) is part of the GISSI Outlier Project, jointly promoted by the Heart Care Foundation Onlus - Italian Association of Hospital Cardiologists (ANMCO) and the Mario Negri Institute of Pharmacological Research in Milan, Italy. It is a prospective, observational, multicentre study aimed at identifying possible new mechanisms promoting or protecting against atherothrombosis. In its observational part, the cross-sectional design allows a comparison of clinical, biomolecular, and imaging characteristics of participants; in its longitudinal phase, subjects are being followed for ≥5 years (5).

### Patients Enrolment

Subjects aged 45 to 75 years, enrolled from January 2011 to June 2013 in the participating centers (see Appendix for detailed information), had to be without any previous clinical manifestations of ischemic heart disease (acute myocardial infarction, unstable angina, chronic stable angina, previous percutaneous or surgical coronary revascularisation, heart failure) undergoing 64-slice (or superior) CCTA in the outpatient clinics of 11 participating centers for suspected CAD.

Main indications for CCTA included: (a) uninterpretable, equivocal, or contraindicated functional stress test; (b) new-onset chest pain syndrome at low-intermediate pre-test likelihood of CAD, and (c) other, including evaluation before valve or non-cardiac surgery, elevated risk profile, arrhythmias, or atypical symptoms. Exclusion criteria were: (a) sub-standard CCTA quality; (b) documented cardiovascular (CV) disease (infarction, angina, revascularization, heart failure); (c) other heart disorders, documented previously or identified at CCTA, such as dilated or obstructive cardiomyopathy, atrial fibrillation, myocarditis, and inflammatory vascular disease; (d) documented peripheral vascular disease (stroke, transient ischemic attack, claudication, revascularisation); and (e) active inflammatory or neoplastic disease.

### Risk Factor Definition

The conventional RFs based on the Adult Treatment Panel III (8) and the 2013 American College of Cardiology/American Heart Association (AHA) guidelines for cardiovascular prevention (9) were applied, as follows: family history of ischaemic heart disease (manifestation of disease in one or more first-degree relative before 55 years of age if male and 65 years if female), arterial hypertension (history, ongoing treatment, or recent blood pressure values >140/90 mmHg), hypercholesterolemia (total serum cholesterol >5.2 mmol/L, or <5.2 mmol/L with ongoing lipid-lowering medications), diabetes mellitus (fasting plasma glucose levels >7.0 mmol/L, or a 2-h value ≥11.1 mmol/L on oral glucose tolerance test or isolated glycated hemoglobin (HbA_1c_) ≥48 mmol/mol or current use of insulin or oral glucose-lowering medications), and cigarette smoking (current or within 1 year). Source data for defining RFs were physical examination, medical records and laboratory tests reported before CCTA; a centrally performed biomarker profile including lipids and metabolic markers allowed a refined assessment of RFs such as diabetes and dyslipidaemia after enrolment. According to the literature, patients with no RFs or a single RF belong to a risk group with <10% risk of events at 10 years according to the Framingham Study, whereas patients with 3 or more RFs belong to a risk group of >20% of events at 10 years (8).

### Coronary Computed Tomography Angiography (CCTA) Analysis

The CCTA data interpretation was performed by advanced plaque assessment using a vessel analysis software with a dedicated tool for plaque volume semiautomatic quantification (PlaqID of CardIQ Xpress 2.0 Package; GE Healthcare, Milwaukee, Wisconsin). In this software, a 3-dimensional image reconstruction including volume rendering and curved multiplanar reformation allows the quantification of coronary plaque volume. Images were independently evaluated by two readers with expertise in cardiovascular imaging. Coronary plaques were defined as structures of at least 1mm^2^ area within or adjacent to the vessel lumen, clearly distinguishable from it and surrounded by pericardial tissue; tissue with signal intensity below −40 Hounsfield units (HU) was considered pericardial fat and excluded from analysis. Coronary arteries were divided into 16 segments according to the AHA classification (9). Normal coronary arteries were defined as no atherosclerotic plaque detected in any segment within the arterial wall or lumen. For each segment, lumen was measured and graded as normal, non-obstructive plaque (<50%), or moderate/severe stenosis (≥50%). High-risk plaque features were also assessed and defined as described in a previous report from CAPIRE (5), and global atherosclerosis burden was assessed on a per patient basis and summarized as a CT-adapted Leaman score as previously reported (10).

#### Patient Groups

Based on the CCTA results, 544 enrolled subjects were grouped into CAD^−^ (clean arteries) and CAD^+^ (atherosclerosis in >5 of 16 segments [segment involvement score >5]), with or without lumen stenoses). The 5-coronary-segment involvement cut-off was chosen to define CAD based on previously assessed prognostic values and on the results of the COronary CT Angiography EvaluatioN For Clinical Outcomes: An InteRnational Multicenter Registry (CONFIRM) study (11). These CAD^+^ and CAD^−^ categories were further divided into those with low RF (RF^−^/CAD^−^ and RF^−^/CAD^+^) or high RF (RF^+^/CAD^+^ and RF^+^/CAD^−^) profiles (Supplementary Figure 1). The RF^+^/CAD^−^ (n = 120) and RF^−^/CAD^+^ (n = 93) individuals were posited as the outlier groups.

### Laboratory Determinations

Peripheral venous blood was drawn, with few exceptions, after an overnight fast. After centrifugation, 0.5 mL serum or plasma aliquots were stored at −70°C in a dedicated biological bank (SATURNE-1; Mario Negri Institute of Pharmacological Research, Milan, Italy). Biomarkers were measured centrally in batches, by personnel blinded to clinical data. Serum creatinine, HbA_1c_ and lipids were measured by standard, automated methods. High-sensitivity C-reactive protein (hsCRP) was measured by an automatic immunoturbidimetric method (Beckman-Coulter, Galway, Ireland); high-sensitivity cardiac troponin T (hs-cTnT) was measured on an automated platform (ECLIA Cobas e411; Roche Diagnostics, Rotkreutz, Switzerland) with a lower detection limit of 3 ng/L.

### Quantification of Plasma Proteins

Protein quantification was performed by modified aptamers, as previously described (12, 13). Briefly, each of ~5,000 individual proteins have its own binding reagent made of chemically modified DNA, referred to as modified aptamer. Each plasma sample is incubated with the mixture of modified aptamers to generate modified aptamer-protein complexes. Unbound modified aptamers and unbound or non-specifically bound proteins are eliminated by 2 bead-based immobilization steps and competition with unlabelled polyanion. After eluting the modified aptamers from the target protein, the fluorescently labeled modified aptamers are directly quantified on an Agilent hybridization array (Agilent Technologies). Calibrators are included so that the degree of fluorescence is a quantitative reflection of the availability of the 3-dimensional shape-charge epitope on each specific protein. Results are expressed as fluorescence intensity units (FU).

### Ethical Statement

The study complies with the Declaration of Helsinki and was approved by locally appointed ethics committees; written informed consent was obtained from all patients.

### Statistical Analysis

Continuous variables are presented as mean ± standard deviation (SD); variables with a skewed distribution (by the Shapiro-Wilk test) are given as median [interquartile range]; the latter were log-transformed for use in parametric testing. Group values were compared by the Wilcoxon test, proportions by the X^2^ test; ANCOVA was used to adjust group comparisons for covariates.

Two-way partial least square (PLS) was employed to rank proteins according to the strength of their separate association with RFs or CAD. This method has been shown to be preferable to random forest or least absolute shrinkage and selection operator (Lasso) regression when the number of predictors (e.g., proteins) is much larger than the number of cases and when there is a high degree of potential multicollinearity in the data (14). Proteins were ranked according to the Variable Importance in the Projection (VIP) score. While a VIP score >2 or >1 is generally considered sufficient, we used a more selective criterion, *i.e.*, a VIP score >3, to enhance the strength of association with RFs or CAD (15).

Proteins' ability to predict phenotypic grouping was assessed by receiver operating characteristic area-under-curve (AUC_ROC_) plots. Principal Component Analysis (PCA) was performed on correlations using a Varimax factor rotation. Multivariate logistic regression was carried out by standard methods. R and SPSS-IBM for Mac Os X software were used; the statistical significance threshold level was set at *p* < 0.05, adjusted for multiple comparisons as appropriate.

## Results

The demographic, clinical and metabolic characteristics of the study cohort were generally consistent with the predefined grouping (Tables 1, 2). Of note, a higher prevalence of male sex, older age, and higher body mass index (BMI) characterized both CAD^+^ groups as compared to the CAD^−^ groups. Most differences between the 4 groups (except for BMI, waist girth, current blood pressure, HbA_1c_, and eGFR) were also present between the RF^+^/CAD^−^ and RF^−^/CAD^+^ outlier groups, reflecting the *a priori* criteria used to define them. Thus, RF^−^/CAD^+^ outliers had a significantly lower prevalence of hypertension, hypercholesterolemia, diabetes, smoking and family history of ischemic heart disease. High density lipoprotein (HDL)-cholesterol was higher, and hsTnT lower, in the RF^+^/CAD^−^ compared to the RF^−^/CAD^+^ group, as previously described (5).

**Table 1:** Demographics and clinical characteristics of the four study groups^#^.

	**CAD** ^**−**^ **2**		**CAD**^**+**^ **4**		***p**°*****	p^*^
	**RF^**−**^/CAD^**−**^*n =* 229**	**RF^**+**^/CAD^**−**^*n =* 120**	**RF^**−**^/CAD^**+**^ *n =* 93**	**RF^**+**^/CAD^**+**^ *n =* 102**		
Sex (% women)	51	57	10	29	<0.0001	<0.0001
Age (years)	58 ± 9	58 ± 8	64 ± 7	63 ± 7	<0.0001	<0.0001
BMI (kg/m^2^)	25.3 ± 3.7	27.4 ± 3.9	27.3 ± 4.2	28.2 ± 4.6	<0.0001	ns
Waist girth (cm)	89 ± 19	94 ± 19	92 ± 29	97 ± 22	0.0128	ns
Familial IHD (%)	14	65	10	59	<0.0001	<0.0001
Hypertension (%)	24	86	38	91	<0.0001	<0.0001
Hypercholesterolaemia (%)	29	94	30	94	<0.0001	<0.0001
Diabetes (%)	0	24	0	38	<0.0001	<0.0001
Current smoking (%)	7	46	9	54	<0.0001	<0.0001
Cigarettes (n°/day)	0.7 ± 3.1	7.0 ± 9.5	1.4 ± 4.8	9.4 ± 13.2	<0.0001	<0.0001
Systolic BP (mmHg)	125 ± 14	129 ± 15	131 ± 16	135 ± 17	<0.0001	ns
Diastolic BP (mmHg)	78 ± 8	78 ± 8	80 ± 8	81 ± 8	0.0045	(0.08)
eGFR (mL/min^−1.^1.73m^−2^)	91 ± 12	89 ± 13	85 ± 16	88 ± 14	0.0111	ns

*# Entries are mean ± SD; p°, all groups; p*

* *, outlier groups only (RF+/CAD- vs. RF-/CAD+). BMI, body mass index; BP, blood pressure; CAD, coronary artery disease; eGFR, estimated glomerular filtration rate; IHD, ischaemic heart disease; ns, non significant; RF, risk factor. These CAD^+^ and CAD^−^ categories were further divided into those with low RF (RF^−^/CAD^−^ and RF^−^/CAD^+^) or high RF (RF^+^/CAD^+^ and RF^+^/CAD^−^) profiles (Supplementary Figure 1). The RF^+^/CAD^−^ (n = 120) and RF^−^/CAD^+^ (n = 93) individuals were posited as the outlier groups*.

*The shaded columns identify the outlier groups and the column reporting the p for their comparison*.

**Table 2:** Metabolic parameters of the four study groups^#^.

	**CAD** ^**−**^ **2**		**CAD**^**+**^ **4**		***p**°*****	p^*^
	**RF^**−**^/CAD^**−**^**	**RF^**+**^/CAD^**−**^**	**RF^**−**^/CAD^**+**^**	**RF^**+**^/CAD^**+**^**		
HbA_1c_ (%)	5.31 ± 0.46	5.53 ± 0.79	5.37 ± 0.50	5.98 ± 0.97	<0.0001	ns
LDL-cholesterol (mmol/L)	3.15 ± 0.88	3.49 ± 0.93	3.15 ± 0.90	2.87 ± 0.96	0.0093	ns
HDL-cholesterol (mmol/L)	1.42 ± 0.41	1.34 ± 0.39	1.16 ± 0.31	1.19 ± 0.31	<0.0001	0.002
Triglycerides (mmol/L)	0.85 [0.63]	1.29 [1.27]	1.26 [0.88]	1.33 [0.99]	<0.0001	ns
non-HDL-cholesterol (mmol/L)	3.57 [1.19]	3.82 [1.58]	3.85 [1.29]	3.33 [1.58]	0.0098	ns
hsCRP (mg/dL)	0.12 [0.23]	0.17 [0.35]	0.15 [0.39]	0.24 [0.50]	0.0003	ns
hsTnT (ng/L)	4.75 [4.78]	4.80 [4.03]	6.89 [4.67]	6.48 [5.00]	<0.0001	<0.0001

*# Entries are mean ± SD or median [interquartile range]; p°, all groups; p*

* *, outlier groups only (RF+/CAD- vs RF-/CAD+). CAD, coronary artery disease; CRP, C-reactive protein; HbA1c, glycated hemoglobin; HDL, high-density lipoprotein; hs, high sensitivity; LDL, low-density lipoprotein; ns, non significant; RF, risk factors; TnT, troponin T*.

*The shaded columns identify the outlier groups and the column reporting the p for their comparison*.

**Figure 1:**
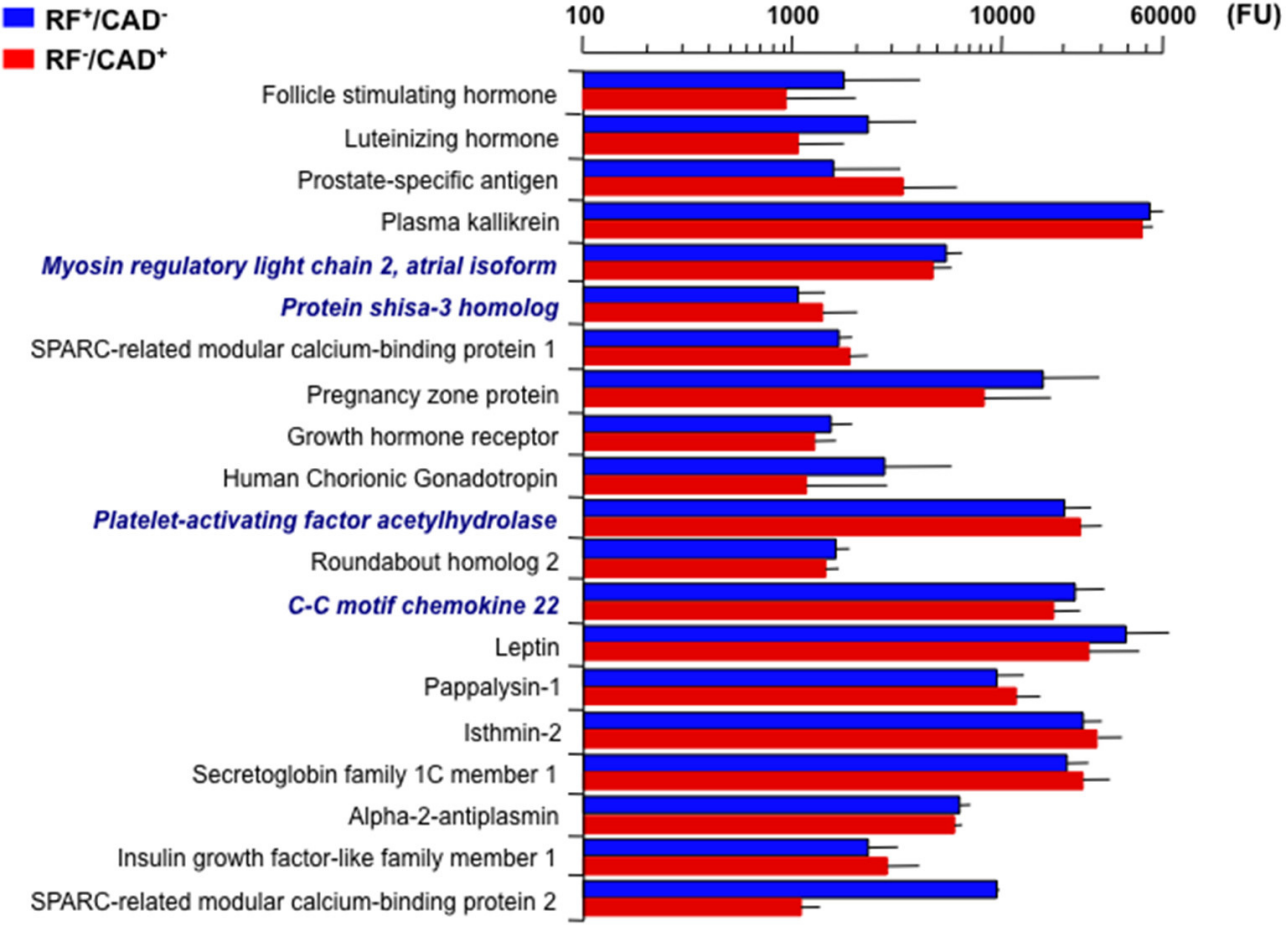
Twenty top plasma signals associated with the outlier groups (CAD^+^/RF^−^ and CAD^−^/RF^+^). Signals are in fluorescence units (FU). Bars are mean ± standard deviation. Bold italics indicate the four proteins that remained significantly different between the two groups after adjusting for gender and age. CAD, coronary artery disease; RF, risk factor.

The association of proteins with the clinical data was explored in a stepwise manner. First, the PLS analysis was restricted to the two outlier groups. Here, 20 proteins were found to significantly differ between them, *i.e*., they achieved a VIP score ≥3 (Figure 1). Because gender and age differed considerably between these groups (Table 1), the corresponding between-group differences in individual protein levels were adjusted for gender and age in separate ANCOVA models.

Consequently, only four proteins retained statistical significance: myosin regulatory light chain 2 atrial isoform (MYO) and C-C motif chemokine 22 (C-C22) were lower (4530 ± 104 vs. 5169 ± 92, *p* = 8.5e^−4^ and 17271 ± 624 vs. 21784 ± 780, *p* = 4.4e^−2^, respectively), and protein shisa-3 homolog (PS-3) and platelet-activating factor acetyl-hydrolase (PAF-AH) were higher (1345 ± 70 vs. 1043 ± 30, *p* = 2.1e^−2^ and 23065 ± 635 vs. 19484 ± 559, *p* = 4.4e^−2^), in RF^−^/CAD^+^ as compared to RF^+^/CAD^−^ (Figure 1).

**Figure 2:**
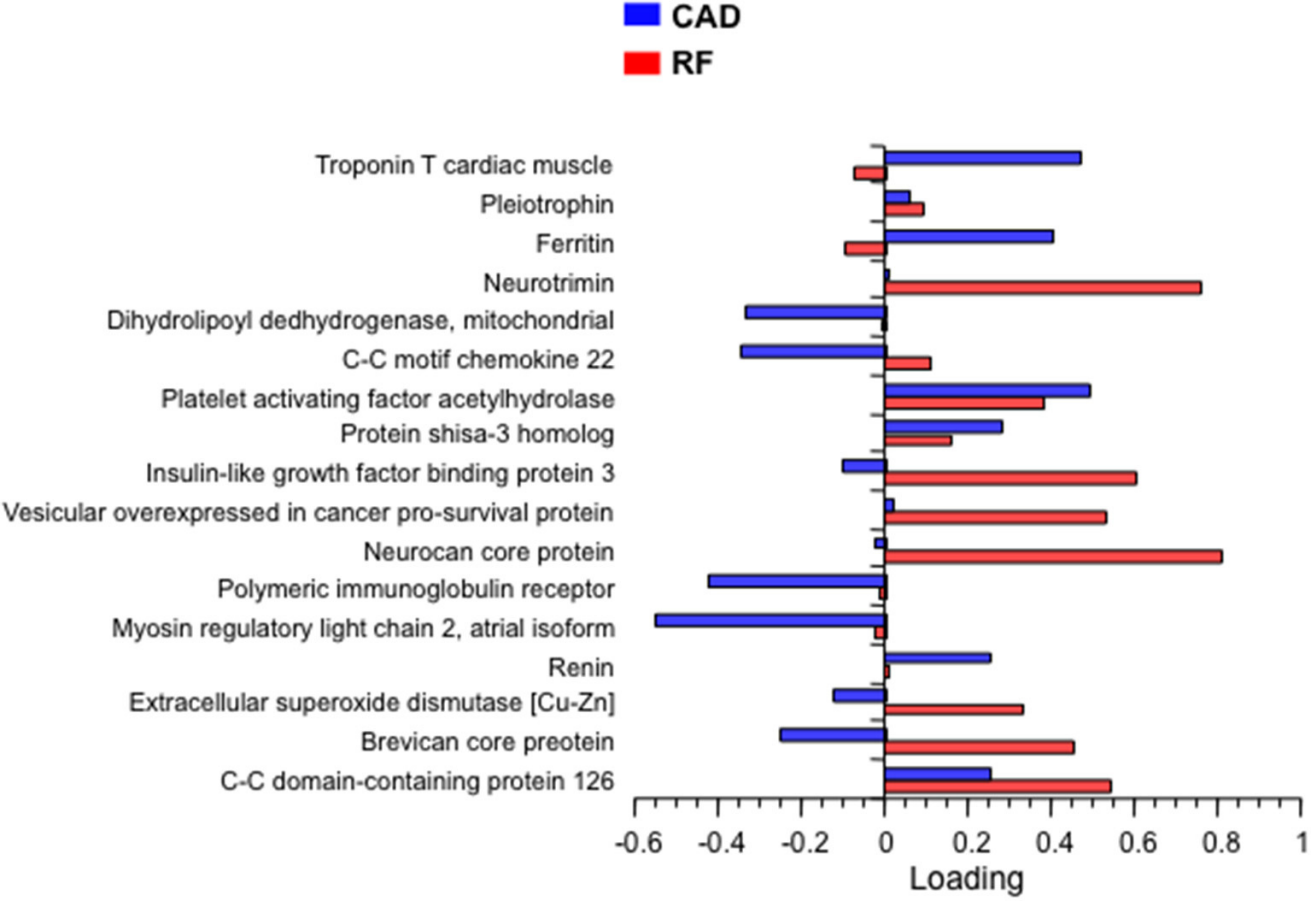
Principal component loading of the 17 proteins in Table 3 on RFs and CAD. CAD, coronary artery disease; RF, risk factor.

**Figure 3:**
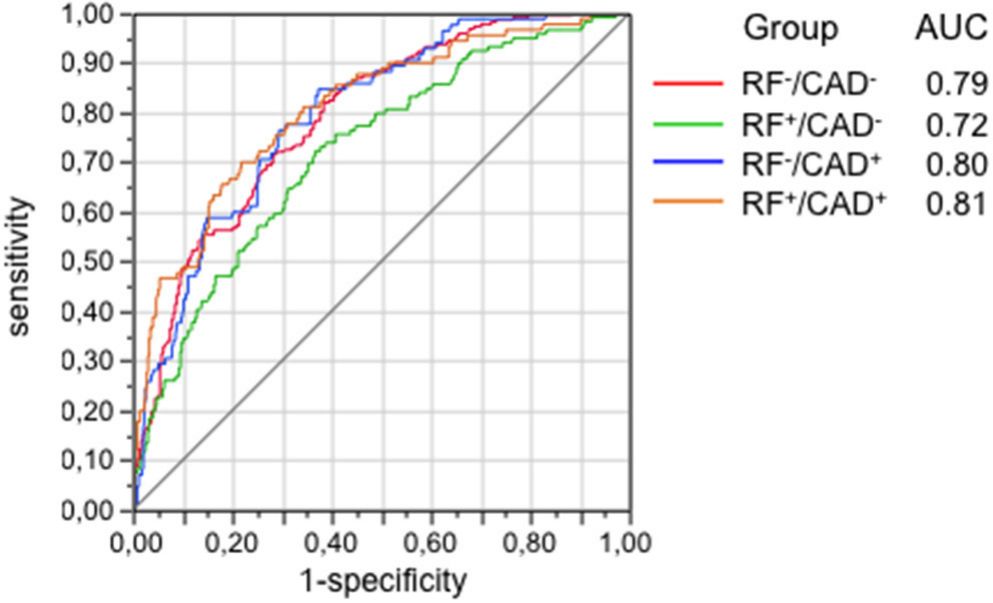
Ability of the 12 proteins selected from Table 3 to predict groups as tested by the area-under-curve (AUC) of the receiver operating characteristics (ROC) functions. Protein signals were standardized to the median and log-transformed. CAD, coronary artery disease; RF, risk factor.

In the next step, bivariate PLS was used on the entire database, with RFs and CAD as the dichotomic response variables. Using again the restrictive criterion of a VIP value ≥3, 13 proteins topped the list (Table 3). Following adjustment for gender, age and multiplicity, four proteins (polymeric immunoglobulin receptor (PIR), neurocan core protein (NCP), vesicular overexpressed-in-cancer pro-survival protein (Ves), and insulin-like growth factor binding protein 3 (IGFBP3) remained significantly associated with the presence of RFs only, 1 [brevican core protein (BCP)] with the presence of CAD only, and 3 proteins [coiled-coil domain-containing protein 126 (C-C126), extracellular superoxide dismutase [Cu-Zn] (SOD), and renin] with the presence of both RFs and CAD. In addition, in this analysis the four proteins previously identified from the direct comparison of the outlier groups retained statistical significance, three of them (PS-3, PAF-AH, and C-C22) in association with RFs only, and one (MYO) in association with CAD alone. The direction and strength of association of each of the 17 proteins in Table 3 were tested by principal component analysis and are shown in Figure 2 as loadings on the two dichotomic factors, RFs and CAD. Thus, by restricting statistical significance to *p* values <0.001, 12 out of ~5,000 proteins were associated with the 2 pre-set discriminants of the four study groups, namely RFs and CAD, independently of the main anthropometric determinants (gender and age). The strength of this panel of proteins was tested in a logistic model where the response variable was the study group, and the 12 proteins were the predictors. As shown in Figure 3, the AUC_ROC_ ranged from 0.72 for the RF^+^/CAD^−^ group to 0.81 for the RF^+^/CAD^+^ group, with an overall *r* value of 0.47 and a *p* value of 1.0e^−38^.

**Table 3:** Plasma proteins independently associated with cardiovascular risk factors (RF) and coronary artery disease (CAD)^*^.

	**RF^**−**^/CAD^**−**^**	**RF^**+**^/CAD^**−**^**	**RF^**−**^/CAD^**+**^**	**RF^**+**^/CAD^**+**^**	***p_***RF***_***	***p_***CAD***_***
*Dihydrolipoyl dehydrogenase, mitochondrial*	*6.39 ± 4.01*	*6.01 ± 2.04*	*5.74 ± 2.60*	*5.29 ± 1.35*	*4.3e-2*	*2.5e-2*
Polymeric immunoglobulin receptor	3.75 ± 6.55	4.34 ± 2.68	3.17 ± 1.46	4.53 ± 2.51	**7.0e-9**	ns
Coiled-coil domain-containing protein 126	1.14 ± 0.30	0.97 ± 0.29	0.97 ± 0.25	0.92 ± 0.30	**2.6e-7**	**7.8e-5**
*Neurotrimin*	*6.08 ± 1.32*	*5.98 ± 1.48*	*5.34 ± 1.18*	*5.43 ± 1.31*	*ns*	*ns*
*Ferritin*	*9.37 ± 8.21*	*8.50 ± 6.72*	*12.6 ± 0.83*	*11.4 ± 0.80*	*ns*	*ns*
Neurocan core protein	4.19 ± 1.46	3.36 ± 1.40	3.39 ± 1.25	3.09 ± 1.11	**3.3e-8**	*1.9e-2*
*Pleiotrophin*	*15.4 ± 20.4*	*12.2 ± 3.92*	*17.8 ±17.7*	*19.0 ± 34.1*	*2.1e-2*	*9.9e-3*
Vescicular overexpressed in cancer pro survival protein	2.16 ± 0.67	1.92 ± 0.63	2.03 ± 1.90	1.80 ± 0.85	**1.5e-4**	*6.3e-3*
*Troponin T cardiac muscle*	*1.31 ± 1.69*	*1.18 ± 0.42*	*1.41 ± 0.53*	*1.34 ± 0.42*	*ns*	*ns*
Brevican core protein	1.58 ± 0.83	1.48 ± 0.66	1.33 ± 0.44	1.29 ± 0.34	*2.3e-2*	**1.2e-3**
Insulin-like growth factor binding protein 3	16.9 ± 3.14	15.9 ± 3.75	14.8 ± 3.42	14.8 ± 3.57	**1.4e-3**	*3.5e-2*
Extracellular superoxide dismutase [Cu-Zn]	31.2 ± 27.3	24.2 ± 9.86	25.3 ± 21.2	24.6 ± 21.8	**1.7e-3**	**3.2e-4**
Renin	15.2 ± 8.88	17.5 ± 11.2	18.6 ± 10.5	28.1 ± 17.4	**2.2e-4**	**1.6e-4**
Myosin regulatory light chain 2, atrial isoform	5.02 ± 1.18	5.17 ± 1.00	4.50 ± 0.95	5.07 ± 1.04	*4.0e-2*	**1.6e-3**
Protein shisa-3 homolog	1.21 ± 0.56	1.04 ± 0.32	1.32 ± 0.63	1.14 ± 0.56	**4.1e-5**	ns
Platelet activating factor acetylhydrolase	21.7 ± 6.07	19.5 ± 6.10	23.0 ± 5.66	20.2 ± 6.30	**1.3e-5**	ns
C-C motif chemokine 22	18.2 ± 6.24	21.8 ± 8.51	17.3 ± 5.48	20.5 ± 8.91	**1.6e-6**	ns

* * Entries are mean ± SD of FU (fluorescence units, x10^−3^); p_RF_ and p_CAD_, p values for the independent association with RF and CAD, adjusted by gender, age and multiple comparisons. Proteins non-significantly associated with both RF and CAD are in italics. The shaded columns identify the outlier groups; the shaded rows highlight the 4 proteins that discriminate the outlier groups (see also Figure 1). In bold are Bonferroni-adjusted p values*.

To quantify the unique association of each protein with individual clinical parameters independently of the other proteins and of gender, age and BMI, we ran multivariate regression models with each the 12 proteins as the dependent factor and all clinical parameters as independent variables in addition to gender, age and BMI. As shown in Table 4, serum HDL-cholesterol was a strong positive correlate of 6 proteins (C-C126, NCP, Ves, BCP, IGFBP3, and SOD, which were all interrelated), while renin was strongly associated with higher HbA_1c_ and lower eGFR. Of note, PAF-AH was strongly associated with serum non-HDL-cholesterol.

**Table 4:** Independent association of the 12 hit plasma proteins with individual biomarkers (adjusted for sex, age and BMI)^*^.

	**HbA_**1c**_**	**SBP**	**HDL**	**Non-HDL**	**TG**	**hsCRP**	**hsTnT**	**eGFR**
Polymeric immunoglobulin receptor	+	ns	+	ns	ns	+	ns	ns
Coiled-coil domain-containing protein 126	–	ns	+++	–	–	—	ns	–
Neurocan core protein	–	–	++	ns	—	—	ns	ns
Vescicular overexpressed in cancer pro survival protein	ns	ns	+++	–	—	—	ns	ns
Brevican core protein	ns	-	+++	ns	-	—	ns	ns
Insulin-like growth factor binding protein 3	ns	–	+++	ns	–	ns	ns	ns
Extracellular superoxide dismutase [Cu-Zn]	-	ns	+++	ns	-	ns	ns	-
Renin	+++	ns	ns	-	ns	ns	+	—
Myosin regulatory light chain 2, atrial isoform	ns	-	ns	ns	ns	ns	ns	+
Protein shisa-3 homolog	ns	ns	-	ns	–	ns	ns	–
Platelet activating factor acetylhydrolase	–	ns	—	+++	—	–	ns	ns
C-C motif chemokine 22	ns	-	ns	ns	+	ns	ns	ns

* * +++, ++, and +: strong, intermediate, and weak positive association; —, –, and - : strong, intermediate, and weak negative association. BMI, body mass index; eGFR, estimated glomerular filtration rate; HbA_1c_, glycated hemoglobin; HDL, HDL-cholesyterol; hsCRP, high-sensitive C-reactive protein; hsTnT, high-sensitivity troponin-T; Non-HDL, non-HDL cholesterol; ns, non significant; SBP, systolic blood pressure; TG, triglycerides*.

**Figure 4:**
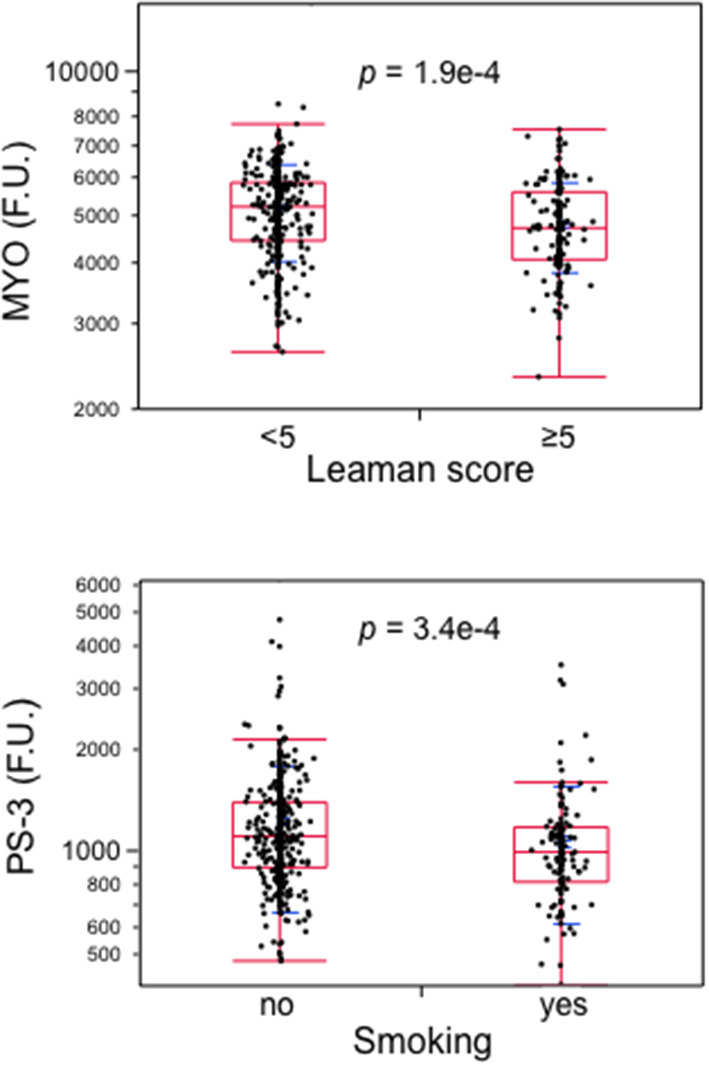
Boxplots of atrial myosin regulatory light chain 2 (MYO) in subjects with a Leaman score of <5 vs. ≥5 and of protein shisa-3 homolog (PS-3) by smoking. Data from the whole cohort; *p*-values by Wilcoxon test. MYO, atrial myosin regulatory light chain 2; PS-3, protein shisa-3 homolog.

The next step was to test the independent association of each of the 12 proteins with the presence of RFs and CAD (Supplementary Table 1). A higher BMI was associated with lower levels of the proteins that were directly related to HDL-cholesterol. Gender also had a strong impact on most of the 12 proteins, as did smoking. Plasma renin was the strongest correlate of CAD. MYO and PS-3 (involved in vascular repair or adverse remodeling) retained independence from BMI, cholesterolemia, hsCRP or HbA_1c_ levels (Table 4, Contribution to the field). By way of example, MYO was reduced not only in the group with CAD and low RFs (RF^−^/CAD^+^) but also in the whole cohort in association with a Leaman Score of ≥5. Conversely, PS-3 was not only reduced in the group with high RFs and no CAD (RF^+^/CAD^−^) but also in association with one of the major RFs, *i.e.*, smoking (Figure 4).

Finally, each of the 12 proteins was tested against the percentage of patients on treatment with the main classes of drugs (while adjusting for gender, age, and BMI). As shown in Supplementary Table 2, drug therapies generally had weak or null associations with protein levels except for some expected link (*e.g.*, higher renin levels in patients using ACEi or ARB). For completeness, Supplementary Tables 3, 4 list the top proteins associated with the extreme groups (CAD^−^/RF^−^ and CAD^+^/RF^+^, respectively).

## Discussion

In the present study we searched for new potential biomarkers by applying high performance proteomics in four different phenotypes, defined according to the presence of traditional cardiovascular RFs and CCTA-assessed CAD. Additionally, linked the resulting “hit” proteins with clinical and biochemical characteristics as well as other risk markers.

Several of the proteins identified in this study have been investigated in human disease and their role in the atherosclerotic process has been assessed; others have only been studied in animal models, with no clear disease correlates. From a general point of view, the twelve hit proteins take part in immune system responses, fibrosis, oxidation, or proliferating/healing signaling pathways, reflecting the complexity of the human proteome applied to CAD.

After adjustment for gender and age, two proteins remained associated with the absence of CCTA-proven CAD despite the presence of multiple RFs, *i.e*., myosin regulatory light chain 2 atrial isoform (MYO or MLC-2a) and C-C motif chemokine 22 (C-C22). Therefore, for these two proteins a protective role against CAD may be hypothesized. Sarcomeric proteins have recently drawn considerable attention as therapeutic targets to improve myocardial function. The recently published results of the Global Approach to Lowering Adverse Cardiac Outcomes through Improving Contractility in Heart Failure (GALACTIC-HF) trial showed that omecamtiv mecarbil, a cardiac myosin activator, reduced the incidence of heart failure hospitalization and cardiovascular death in patients with systolic heart failure compared with placebo (16). MYO is a sarcomeric protein that enhances migration, contractility and inotropism in vascular smooth muscle cells (VSMC) in a calcium-independent fashion (17). Its reduction in the intima of coronary plaques has been shown to wound the healing potential of VSMC (17, 18). In our dataset, the biological strength of this association is confirmed by the reduced MYC levels in individuals with a high Leaman score, *i.e.*, a high burden of atherosclerotic involvement in their coronaries. C-C22 is an immunosuppressant chemotactic peptide, predominantly investigated in cancer biology, which also seems to have a protective role against atherogenesis (19).

In the outlier counterpart (presence of CAD with low levels of RFs), protein Shisa 3 homolog (PS-3) and platelet-activating factor acetyl hydrolase (PAF-AH) were identified as markers of CAD. PS-3 is a scaffold protein mediating the Wnt/beta-catenin signaling pathway (20). Recent data identify PS-3 involvement in pathological adverse vascular remodeling (21), suggesting a detrimental role of PS-3 in CAD development. A large number of studies have identified PAF-AH (also known as lipoprotein-associated phospholipase A2, Lp-PLA_2_) as a biomarker of vascular inflammation and atherosclerosis (22). PAF-AH serves the biologic function of degrading products within oxidized LDL, causing the instability of the atherosclerotic plaque; accordingly, in the current data, PAF-AH was strongly associated with non-HDL-cholesterol (23). Numerous studies have shown its correlation with long-term cardiovascular events in patients with both stable and unstable CAD (22, 24). However, two large randomized, placebo-controlled trials of the PAF-AH inhibitor, darapladib, failed to prove a cardioprotective effect (25, 26), therefore its value as a therapeutic target is uncertain.

Among the four proteins associated with RF^+^/CAD^−^, insulin-like growth factor binding protein 3 (IGFBP3) is the best known (27). This protein binds ~90% of insulin-like growth factors and is upregulated by hypoxia; recently, its suppression by salvianolic acid B has been suggested to improve myocardial function in diabetes-associated cardiac fibrosis (28). Moreover, growth hormone, the stimulus for IGF production and the key regulator of IGFBP3, has been shown to be associated with coronary atherosclerosis, independently of other RFs. Hence, it is not unlikely that IGFBP3 clusters with other cardiovascular RFs, although its role remains disputed (29).

Finally, two proteins associated with the presence of both RFs and CAD have a convergent biologic role. The first one, extracellular superoxide dismutase (Cu-Zn SOD), is a major antioxidant enzyme whose alterations possibly reflect enhanced oxidative stress; moreover, impaired Cu-Zn SOD expression or catalytic activity has been identified in several physiological situations such as aging and age-associated diseases (30). The second one is renin, which was the strongest correlate of CAD in our cohort and whose link with cardiovascular events has a firm pathological basis and is supported by growing clinical evidence: renin is the activator of the renin-angiotensin-aldosterone system, whose pharmacological inhibition reduces morbidity and mortality of patients with CAD (31), and high levels of renin are associated with atherosclerosis, hypertensive cardiomyopathy, and impaired left ventricular function (32). The observational Multiethnic Study of Atherosclerosis reported an independent association between plasma renin activity and cardiovascular outcomes in patients without previous CV events who had angiographically proven CAD or subclinical cardiovascular disease (33).

Our qualitative principal component analysis indicated a clustering of seven of the twelve hit proteins with HDL-cholesterol. It is well established that HDL-cholesterol levels are strongly and inversely correlated with the risk of CAD; however, interventional studies raising HDL levels did not reduce cardiovascular events (34–37). In fact, it is the quality of HDL-cholesterol particles that seems to be relevant, because it affects their functionality not only in the atherosclerotic process but also in immunomodulation and inflammation (38). In line with the pathophysiological relevance of lipid metabolism, several classes of apolipoproteins have been studied as cardiovascular risk factors, and their pathophysiological role in CAD is firmly established (38). Consistently, in the current study the PAF-AH signal was significantly higher in the RF^−^/CAD^+^ group as compared to RF^+^/CAD^−^, and was reciprocally related to HDL-cholesterol. A weak negative association with HDL-cholesterol was also seen for PS-3, the other protein that was higher in the RF^−^/CAD^+^ group and that remains inversely associated with the presence of other RFs. Of all hits, reduced MYO and reduced PS-3 (involved in vascular repair and adverse remodeling) retained their strong associations with CAD^+^/RF^−^ and CAD^−^/RF^+^ phenotypes, respectively, independently of body mass, cholesterolemia, C-reactive protein or glycated hemoglobin levels.

### Limitations

Some limitations have to be taken into account in our study. First of all, definition of patients as RF+ or RF- relied on traditional risk markers and may be approximative, even if we did all efforts to correlate these profiles to a broad set of clinical features. Moreover, selecting 12 out of ~5000 proteins, *i.e.*, filtering out >99% of the proteome, very likely misses potentially important protein signals. Thus, the very stringent statistical conditions we applied slice through just the tip of the iceberg of protein networks biologically related to CAD. Furthermore, hit proteins should be directly quantified to confirm their association with CAD or RFs. By way of example, in our cohort, the aptamer-based signals for hs-CRP and troponin-T, the two best accepted biomarkers of CAD, were well correlated with the corresponding levels from direct assays (Supplementary Figure 2). On the other hand, the use of modified aptamer arrays, a powerful and validated tool (12), allows one to scan a large fraction of the entire circulating proteome (~10,500 proteins, including alternative splicing products). In a previous study of individuals with stable CAD, 200 plasma proteins (out of 1,130 screened by the aptamer technology) were found to be associated with incident cardiac events; of these, 9 were selected to assemble a predictive model (7). The list of prognostic proteins in that study (Supplementary Table 4 in ref. 7) and our hit list do show some overlap of the main functional domains despite the difference in cohort phenotype (stable CAD vs. case mix) and response (CV events vs. CCTA-proven CAD). However, the aim of our analysis was not to improve predictivity as much as to delineate more specific links among individual proteins, atypical clinical phenotypes and biochemical risk factors. Thus, seven of 12 proteins clustered around HDL-cholesterol, which, in turn, appeared to be mediating the effect of obesity; renin seemed to drive worse coronary atherosclerotic involvement in the presence of elevated HbA_1c_ levels and reduced eGFR.

We did not perform any internal validation because of the limited size of the outlier groups prevents from applying reliable statistical procedures. The highly selected CCTA population of CAPIRE and its peculiar design might prevent external validation.

## Conclusion

Thanks to a complete segregation of CAD phenotypes and a reasonably wide separation of risk factor distribution, in the present CAPIRE study proteomic screening *per se* was able to discriminate CAD+/RF– from CAD–/RF+. Correlation analyses linking physiology to proteins, clinical risk factors and biochemical risk markers led to the extraction of MYO and PS-3 as two discriminators, independent of established risk factors or biomarkers. Of note, each of them points to opposing vascular processes (healing vs. adverse remodeling), potentially protecting or predisposing to CAD, which warrants further mechanistic investigation as well as validation in other cohorts.

## Data Availability Statement

The data underlying this article will be shared on reasonable request to the corresponding author.

## Ethics Statement

This study, involving human participants, was reviewed and approved by Istituto Svizzero per gli Agenti Terapeutici, Comitato Etico Centro Cardiologico Monzino-IEO, Comitato Etico Aziendale dell'Azienda Ospedaliera-Universitaria, Comitato Etico Parma, Comitato Etico Modena, Comitato per la Sperimentazione Clinica dei Medicinali, AUSL 12 di Viareggio – Ufficio Ricerca, Segreteria Locale ASUR del CERM Marche. The patients provided their written informed consent to participate in this study.

## Author Contributions

EF wrote the manuscript, researched data, and is the guarantor taking responsibility for the contents of the article. MLM contributed to drafting the manuscript and carried out statistical analyses. GF contributed to drafting the manuscript and managed revision and editing. RL supervised the routine laboratory analyses. SW was responsible for the proteomics. FA, DA, RL, MM, SW, AMas, and AMag critically reviewed and edited the manuscript and contributed significantly to discussion. All authors contributed to the article and approved the submitted version.

## Funding

This work was supported by Heart Care Foundation of the Italian Association of Hospital Cardiologists, Florence, Italy.

## Conflict of Interest

FA reports consultancy/speaker fees from Amgen, Bayer, BMS/Pfizer, and Daiichi Sankyo outside the submitted work. SW is an employee and shareholder of SomaLogic Inc., Boulder, Colorado, USA. AMag reports personal fees from Bayer, Fresenius, and Novartis outside the submitted work. The remaining authors declare that the research was conducted in the absence of any commercial or financial relationships that could be construed as a potential conflict of interest.

## Publisher's Note

All claims expressed in this article are solely those of the authors and do not necessarily represent those of their affiliated organizations, or those of the publisher, the editors and the reviewers. Any product that may be evaluated in this article, or claim that may be made by its manufacturer, is not guaranteed or endorsed by the publisher.

## Supplementary Material

Supplementary Figure 1 Grouping of the study subjects. CAD, coronary artery disease; CCTA, coronary computed tomography angiography; RF, risk factor.

Supplementary Figure 2 Scatterplots of aptamer-based signal *vs* directly assayed plasma concentration of C-reactive protein and troponin-T in the entire cohort. The dark-shaded and light-shaded bands are the fit and individuals 95% confidence interval, respectively. FU, fluorescence units; hs-CRP, high-sensitivity C-reactive protein; hs-cTnT, high-sensitivity cardiac troponin T.

Supplemental Table 1 Independent association of the 12 hit proteins with individual cardiovascular risk factors and coronary artery disease^*^.

Supplemental Table 2 Influence of treatments on plasma proteins independent of RFs and CAD^*^.

Supplemental Table 3 Top proteins associated with the CAD+/RF+ phenotype.

Supplemental Table 4 Top proteins associated with the CAD-/RF- phenotype.
