# Supplementary material for: Differential Proteomics of Cardiovascular Risk and Coronary Artery Disease in Humans
Source: Front Cardiovasc Med. 2022 Feb 4;8:790289. doi: 10.3389/fcvm.2021.790289 (PMC8855064; doi:10.3389/fcvm.2021.790289)
Supplement: Supplementary file 1 [file Data_Sheet_1.doc]

**Appendix**

**Steering Committee**

A. Maseri † (Chairman; Firenze), D. Andreini (Milano), S. Berti (Massa), M. Canestrari (Fano), G. Casolo (Lido di Camaiore), D. Gabrielli (Roma), R. Latini (Milano), M. Magnoni (Milano), P. Marraccini (Pisa), T. Moccetti (Lugano), M.G. Modena (Modena)

**Coordinating Center:** A.P. Maggioni, M. Gorini, F. Bianchini, I. Cangioli, A. Lorimer (Centro Studi ANMCO Firenze)

**Imaging Core Laboratory:** D. Andreini, G. Pontone, E. Conte (Centro Cardiologico Monzino Milano)

**Centralized biobank and biomarker core laboratory**: D. Novelli, F. Gaspari, S. Ferrari, A. Cannata, N. Stucchi, M. Fois, R. Bernasconi, G. Balconi (Istituto Mario Negri, Milano and Bergamo), T. Vago, T. Letizia (Ospedale Luigi Sacco, Milano), B. Bottazzi, R. Leone (Istituto Clinico Humanitas, Rozzano).

**Central ECG Reading:** I. Suliman (Centro Studi ANMCO, Firenze)

**Psychologists CRF Group:** M. Sommaruga † (IRCCS Salvatore Maugeri Unità di Psicologia, Milano), P. Gremigni (Dipartimento di Psicologia Università di Bologna)

**Participating Centers and Investigators**

Fano, Ospedale S Croce (R. Olivieri); Fermo, Ospedale Civile A. Murri (L. Pennacchietti); Lido di Camaiore, Nuovo Ospedale Versilia (M. Magnacca); Lugano, Cardiocentro Ticino (M.G. Rossi, E. Pasotti, T. Moccetti); Massa, FTGM - Stabilimento di Massa (A. Clemente); Milano, Centro Cardiologico Monzino (D. Andreini, G. Pontone, S. Mushtaq); Modena, Ospedale Policlinico (E. Mauro, G. Boriani); Parma, AOU. di Parma (F. Pigazzani); Pisa, AOU Pisana (L. Faggioni); Pisa, FTGM - Stabilimento di Pisa (M. Ciardetti); Udine, AOU SM della Misericordia (M. Puppato)
