# Supplementary material for: Differential Proteomics of Cardiovascular Risk and Coronary Artery Disease in Humans
Source: Front Cardiovasc Med. 2022 Feb 4;8:790289. doi: 10.3389/fcvm.2021.790289 (PMC8855064; doi:10.3389/fcvm.2021.790289)
Supplement: Supplementary file 2 [file Data_Sheet_2.DOCX]

**Institutional Review Board CAPIRE Study**

| **Institutional Review Board** | **Address** | **City** |
| --- | --- | --- |
| Istituto Svizzero per gli Agenti Terapeutici | c/o Swissmedic | Bellinzona |
| Comitato Etico Centro Cardiologico Monzino-IEO | c/o Istituto Europeo Oncologia | Milano |
| Comitato Etico Aziendale dell’Azienda Ospedaliera-Universitaria | c/o AO Santa Maria della Misericordia | Udine |
| Comitato Etico Parma | c/o Azienda Ospedaliera di Parma | Parma |
| Comitato Etico Modena | c/o Azienda Ospedaliera Policlinico Modena | Modena |
| Comitato per la Sperimentazione Clinica dei Medicinali | c/o Azienda USL 1 di Massa e Carrara | Massa |
| Comitato per la Sperimentazione Clinica dei Medicinali | c/o Azienda Ospedaliero-Universitaria Pisana | Pisa |
| AUSL 12 di Viareggio – Ufficio Ricerca | c/o Ospedale Versilia | Lido di Camaiore (LU) |
| Segreteria Locale ASUR del CERM Marche | c/o Azienda Sanitaria Regionale delle Marche | Ancona |
