# Supplementary material for: Differential Proteomics of Cardiovascular Risk and Coronary Artery Disease in Humans
Source: Front Cardiovasc Med. 2022 Feb 4;8:790289. doi: 10.3389/fcvm.2021.790289 (PMC8855064; doi:10.3389/fcvm.2021.790289)
Supplement: Supplementary file 3 [file Data_Sheet_3.docx]

**Contribution to the field**

Atypical clinical presentations are useful models to investigate neglected disease mechanisms.

We hypothesize that large-scale proteomics can help unravel additional cardiovascular risk markers that may predispose to coronary artery disease (CAD) beyond traditional risk factors (RF).

In this observational study, more than 5000 proteins were screened in 544 individuals who underwent coronary computed tomography angiography. They were linked to prospectively-defined phenotypes: CAD+ or CAD-, each with either multiple (RF+) or low (RF-) RF profile. Two hits retained independence from body mass, cholesterolemia, C-reactive protein or glycated hemoglobin: reduced atrial myosin regulatory light chain 2 (MYO), involved in vascular repair, with CAD+/RF-, and reduced protein shisa-3 homolog (PS-3), involved in adverse vascular remodeling, with CAD-/RF+.

These data suggest that MYO and PS-3 might be biomarkers for processes leading to CAD independently of established risk factors/biomarkers.
