# Supplementary figures and images for: Differential Proteomics of Cardiovascular Risk and Coronary Artery Disease in Humans
Source: Front Cardiovasc Med. 2022 Feb 4;8:790289. doi: 10.3389/fcvm.2021.790289 (PMC8855064; doi:10.3389/fcvm.2021.790289)

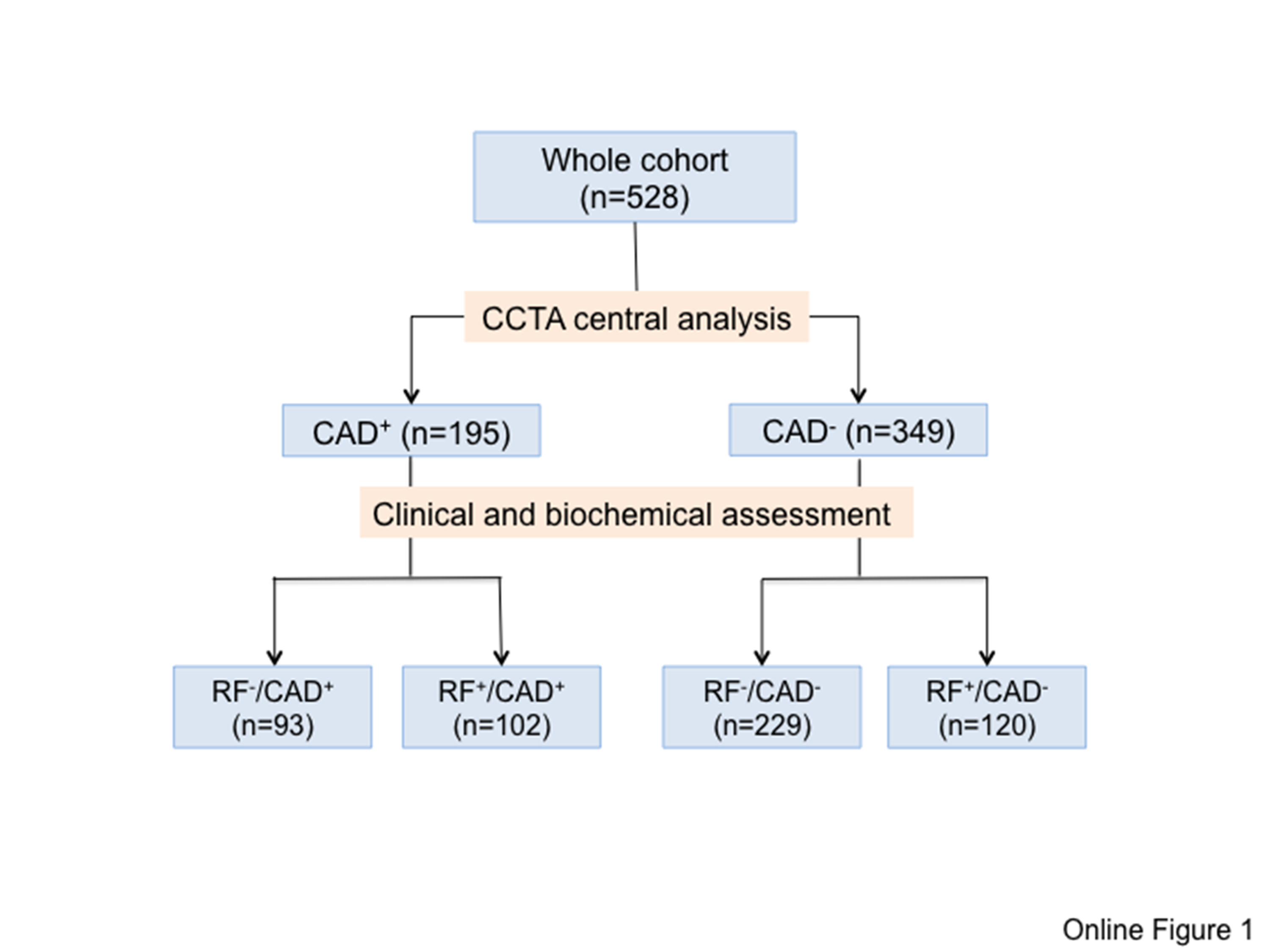

Supplement: Supplementary Figure 1 — Grouping of the study subjects. CAD, coronary artery disease; CCTA, coronary computed tomography angiography; RF, risk factor. [file Image_1.TIFF]

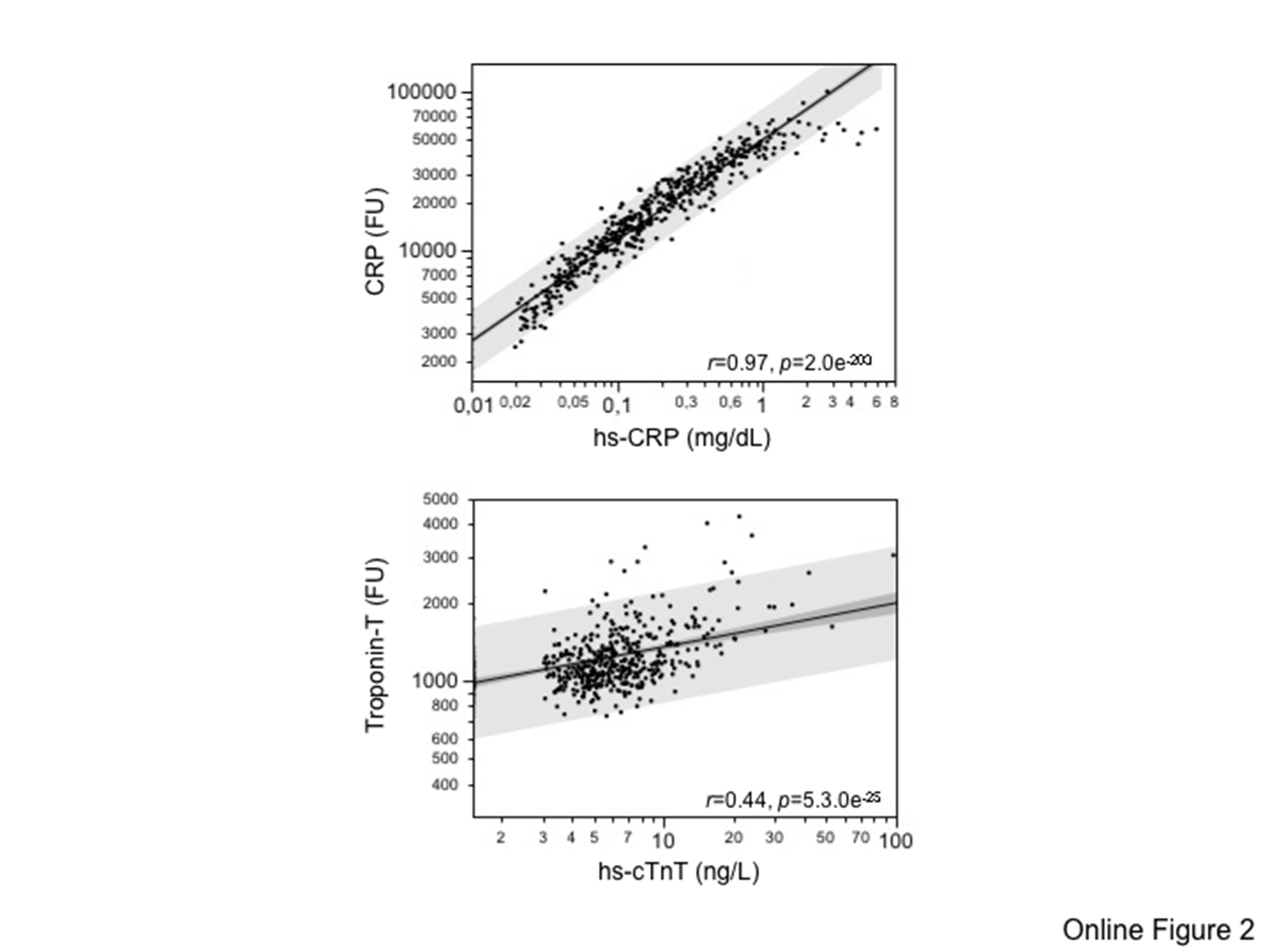

Supplement: Supplementary Figure 2 — Scatterplots of aptamer-based signal vs directly assayed plasma concentration of C-reactive protein and troponin-T in the entire cohort. The dark-shaded and light-shaded bands are the fit and individuals 95% confidence interval, respectively. FU, fluorescence units; hs-CRP, high-sensitivity C-reactive protein; hs-cTnT, high-sensitivity cardiac troponin T. [file Image_2.TIFF]
