# Supplementary material for: Differential Proteomics of Cardiovascular Risk and Coronary Artery Disease in Humans
Source: Front Cardiovasc Med. 2022 Feb 4;8:790289. doi: 10.3389/fcvm.2021.790289 (PMC8855064; doi:10.3389/fcvm.2021.790289)
Supplement: Supplemental Table 1 — Independent association of the 12 hit proteins with individual cardiovascular risk factors and coronary artery disease*. [file Table_1.DOCX]

**Supplemental Table 1 – Independent association of the 12 hit proteins with individual cardiovascular risk factors and coronary artery disease.***

|  | **Male** | **Age** | **BMI** | **FH** | **Smok.** | **HT** | **HChol** | **Diab.** | **CAD** |
| --- | --- | --- | --- | --- | --- | --- | --- | --- | --- |
| Polymeric immunoglobulin receptor | -- | ns | ns | ns | --- | ns | ns | + | ns |
| Coiled-coil domain-containing protein 126 | + | - | --- | ns | -- | ns | - | ns | -- |
| Neurocan core protein | -- | ns | --- | ns | - | ns | - | ns | ns |
| Vescicular overexpressed in cancer pro survival protein | - | ns | --- | + | ns | ns | ++ | ns | ns |
| Brevican core protein | ns | ns | --- | ns | - | ns | ns | ns | - |
| Insulin-like growth factor binding protein 3 | --- | -- | --- | ns | -- | ns | ns | ns | ns |
| Extracellular superoxide dismutase [Cu-Zn] | ns | ++ | ns | ns | - | ns | + | ns | - |
| Renin | ++ | + | ns | ns | ++ | ns | ns | +++ | +++ |
| Myosin regulatory light chain 2, atrial isoform | ns | - | ns | ns | ns | ns | ns | ns | -- |
| Protein shisa-3 homolog | ns | ns | ns | ns | -- | ns | ns | ns | ns |
| Platelet activating factor acetylhydrolase | +++ | ns | -- | ns | ++ | ns | -- | -- | ns |
| C-C motif chemokine 22 | -- | ns | ns | ns | +++ | ns | ns | ns | ns |

# * +++, ++, and + = strong, intermediate, and weak positive association; ---, --, and - = strong, intermediate, and weak negative association.

# BMI = body mass index; CAD = coronary artery disease; Diab. = diabetes; FH = family history of ischemic heart disease; HT = hypertension; Hchol = hypercholesyterolemia;. ns = non significant.
