# Supplementary material for: Differential Proteomics of Cardiovascular Risk and Coronary Artery Disease in Humans
Source: Front Cardiovasc Med. 2022 Feb 4;8:790289. doi: 10.3389/fcvm.2021.790289 (PMC8855064; doi:10.3389/fcvm.2021.790289)
Supplement: Supplemental Table 2 — Influence of treatments on plasma proteins independent of RFs and CAD*. [file Table_2.DOCX]

**Supplemental Table 2 – Influence of treatments on plasma proteins independent of RFs and CAD.***

|  | **ASA**  **(23%)** | **Diu**  **(9%)** | **CaAn**  **(4%)** | **ARB**  **(16%)** | **ACEi**  **(19%)** | **ß-block**  **(25%)**  **(** | **Stat**  **(31%)** |
| --- | --- | --- | --- | --- | --- | --- | --- |
| Polymeric immunoglobulin receptor | ns | ns | ns | ns | ns | - | ns |
| Coiled-coil domain-containing protein 126 | ns | ns | ns | ns | ns | -- | ns |
| Neurocan core protein | - | ns | ns | ns | ns | ns | + |
| Vescicular overexpressed in cancer pro survival protein | ns | ns | ns | ns | ns | ns | ns |
| Brevican core protein | ns | ns | ns | ns | ns | ns | ns |
| Insulin-like growth factor binding protein 3 | ns | ns | ns | ns | ns | ns | ns |
| Extracellular superoxide dismutase [Cu-Zn] | ns | ns | ns | ns | ns | ns | ns |
| Renin | ns | ns | ns | + | + | ns | + |
| Myosin regulatory light chain 2, atrial isoform | ns | ns | ns | ns | ns | ns | ns |
| Protein shisa-3 homolog | ns | ns | ns | ns | ns | ns | ns |
| Platelet activating factor acetylhydrolase | - | ns | ns | - | ns | ns | -- |
| C-C motif chemokine 22 | ns | ns | ns | ns | ns | ns | ns |

# * + = weak positive association; --, and - = intermediate, and weak negative association.

# ACEi =angiotensin-converting enzyme inhibitors; ARB = angiotensin-receptor blockers; ASA = aspirin; ß-block = ß-blockers; CaAn = calcium-channel blockers; CAD = coronary artery disease; Diu = diuretics; ns = non significant; RFs = risk factors; stat = statins.
