# Supplementary material for: Differential Proteomics of Cardiovascular Risk and Coronary Artery Disease in Humans
Source: Front Cardiovasc Med. 2022 Feb 4;8:790289. doi: 10.3389/fcvm.2021.790289 (PMC8855064; doi:10.3389/fcvm.2021.790289)
Supplement: Supplemental Table 3 — Top proteins associated with the CAD+/RF+ phenotype. [file Table_3.DOCX]

**Supplemental Table 3** – Top proteins associated with the CAD+/RF+ phenotype.

| **Protein** | **CAD+/RF+ *vs* All** |
| --- | --- |
| Renin | 6.69 |
| Osteomodulin | 4.8 |
| Growth/differentiation factor 15 | 4.58* |
| Matrix-remodeling-associated protein 8 | 4,57* |
| Heparan-sulfate 6-O-sulfotransferase 3 | 4.56 |
| Adiponectin | 4.26 |
| Leucine-rich repeat-containing protein 15 | 4.2 |
| IGF-binding protein complex acid labile subunit | 3.97 |
| ADAMTS-like protein 2 | 3.91 |
| Macrophage metalloelastase | 3.85 |
| Syntenin-2 | 3.78 |
| Syntenin-1 | 3.73 |
| Amyloid-like protein 1 | 3.68 |
| Epidermal growth factor receptor | 3.68 |
| Serine protease HTRA1 | 3.67 |
| Chondroadherin | 3.66 |
| Neurocan core protein | 3.63 |
| Secretogranin-3 | 3.53 |
| Seizure 6-like protein | 3.49 |
| CUB domain-containing protein 1 | 3.39 |
| Desmoglein-2 | 3.37 |
| Angiotensinogen | 3.35 |
| Oligodendrocyte-myelin glycoprotein | 3.35 |
| Protein S100-A9 | 3.34 |
| Prostaglandin reductase 1 | 3.29 |
| Coagulation factor IX | 3.27 |
| Tetranectin | 3.25 |
| Coagulation factor IXab | 3.21 |
| Tubulointerstitial nephritis antigen-like | 3.2 |
| Sialic acid-binding Ig-like lectin 7 | 3.17 |
| Tissue factor pathway inhibitor | 3.17 |
| Coiled-coil domain-containing protein 126 | 3.14* |
| Cartilage acidic protein 1 | 3.11 |
| Ephrin type-A receptor 4 | 3.1 |
| K voltage-gated channel subfamily E regulatory ßsubunit 5 | 3.1 |
| Dihydropyrimidinase-related protein 2 | 3.09 |
| Contactin-1 | 3.06 |
| Beta-defensin 124 | 3.04 |
| Anthrax toxin receptor 2 | 3.03 |

CAD = Coronary artery disease; RF = risk factors.
