# Supplementary material for: Differential Proteomics of Cardiovascular Risk and Coronary Artery Disease in Humans
Source: Front Cardiovasc Med. 2022 Feb 4;8:790289. doi: 10.3389/fcvm.2021.790289 (PMC8855064; doi:10.3389/fcvm.2021.790289)
Supplement: Supplemental Table 4 — Top proteins associated with the CAD-/RF- phenotype. [file Table_4.DOCX]

**Supplemental Table 4** – Top proteins associated with the CAD-/RF- phenotype.

| **Protein** | **CAD-/RF- *vs* All** |
| --- | --- |
| Fructose-bisphosphate aldolase C | 4.13 |
| Poly [ADP-ribose] polymerase 11 | 3.86 |
| DnaJ homolog subfamily B member 9 | 3.61 |
| Killer cell immunoglobulin-like receptor 3DL1 | 3.55 |
| Glycerol-3-phosphate dehydrogenase [NAD(+)], cytoplasmic | 3.53 |
| C-C motif chemokine 22 | 3.49 |
| NADPH--cytochrome P450 reductase | 3.43 |
| Fatty acid-binding protein, heart | 3.42 |
| Prostate-specific antigen | 3.3 |
| Steroidogenic acute regulatory protein, mitochondrial | 3.19 |
| Serum albumin | 3.15 |
| Apolipoprotein C-III | 3.1 |
| Ecto-ADP-ribosyltransferase 3 | 3.07 |
| HLA class II histocompatibility antigen, DR beta 3 chain | 3.06 |
| Interleukin-18 receptor accessory protein | 3.05 |
| Liver-expressed antimicrobial peptide 2 | 3.03 |
| Coagulation Factor XI | 3.0 |

CAD = Coronary artery disease; RF = risk factors.
